# Blood Eosinophil Count as a Predictive Biomarker of Chronic Obstructive Pulmonary Disease Exacerbation in a Real-World Setting

**DOI:** 10.1155/2023/3302405

**Published:** 2023-05-25

**Authors:** Moegi Komura, Tadashi Sato, Yohei Suzuki, Hitomi Yoshikawa, Naoko Arano Nitta, Mika Hayashi, Eriko Kuwasaki, Kimiko Horikoshi, Toshihiko Nishioki, Mikiko Mori, Yuzo Kodama, Shinichi Sasaki, Kazuhisa Takahashi

**Affiliations:** ^1^Department of Respiratory Medicine, Juntendo University Graduate School of Medicine, 3-1-3 Hongo, Bunkyo-Ku, Tokyo 113-8431, Japan; ^2^Department of Respiratory Medicine, Juntendo University Urayasu Hospital, 2-1-1 Tomioka, Urayasu, Chiba 273-0021, Japan

## Abstract

**Introduction:**

Chronic obstructive pulmonary disease (COPD) is the third leading cause of death, and COPD exacerbation worsens the prognosis. Eosinophilic airway inflammation is a COPD phenotype that causes COPD exacerbation and is correlated with peripheral blood eosinophil count. We analyzed real-world data of COPD patients to assess the risk factors of COPD exacerbation focusing on blood eosinophils.

**Materials and Methods:**

Patients with COPD who visited our hospital between January 1, 2018, and December 31, 2018, were recruited, and their background information, spirometry data, laboratory test results, and moderate-to-severe exacerbation events during the one-year follow-up period were collected from the electronic medical records and analyzed. The COPD exacerbation risk factors were assessed using univariate and multivariate logistic regression analyses.

**Results:**

Twenty-two of 271 (8.1%) patients experienced moderate-to-severe exacerbation. Patients with exacerbation showed worse pulmonary function, and we found that a high blood eosinophil count (≥350 cells/*μ*L; *p*=0.014), low % FEV1 (<50%; *p*=0.002), increase in white blood cell (≥9000 cells/*μ*L; *p*=0.039), and use of home oxygen therapy (*p*=0.005) were risk factors for future exacerbations. We also found a strong correlation between eosinophil count cut-offs and exacerbation risk (*r* = 0.89, *p* < 0.001). On the other hand, there was no relation between exacerbation risk and inhalation therapy for COPD.

**Conclusion:**

In a real-world setting, peripheral blood eosinophil count could be a predictor of future COPD exacerbation.

## 1. Introduction

Chronic obstructive pulmonary disease (COPD) is the third leading cause of death worldwide [1]. In the Global Initiative for chronic obstructive pulmonary disease (GOLD) statement, COPD exacerbation is defined as “an acute worsening of respiratory symptoms that results in additional therapy,” and it causes the deterioration of quality of life, the decline of respiratory functions, and worsens prognosis [2–5]. There are various phenotypes of COPD, one of which is eosinophilic airway inflammation. According to the literature, 30%–40% of COPD patients have eosinophilic airway inflammation [6–9], which is thought to correlate with peripheral blood eosinophil count [10, 11]. High blood eosinophil levels in the disease-stable period are associated with a higher risk of exacerbation [12–14], and post hoc analyses of randomized clinical trials reported prevention of COPD exacerbation by using inhaled corticosteroids (ICS) among highly eosinophilic patients [15–17].

Nowadays, peripheral blood eosinophil count is one of the therapeutic indications for ICS in COPD patients. In the GOLD statement, ICS should be considered in Group D with an eosinophil count ≥300 cells/*μ*L, or in frequent exacerbators with an eosinophil count ≥100 cells/*μ*L. Thus, peripheral blood eosinophil count has been revealed to facilitate deciding the indication for ICS. In contrast, the usefulness of blood eosinophil count for predicting COPD exacerbation is controversial. Recent studies reported that blood eosinophil count can be a useful predictor of future exacerbations [18–20]. Nevertheless, real-world evidence of blood eosinophil count as a predictor of COPD exacerbation is insufficient. In a real-world setting, patients with COPD show various clinical courses and have many comorbidities that affect the clinical course. Therefore, the aim of this study was to assess the risk factors of COPD exacerbation in a real-world setting and confirm whether blood eosinophil count has a predictive role as a biomarker of COPD exacerbation.

## 2. Materials and Methods

The study was conducted in accordance with the guidelines of the Declaration of Helsinki and approved by the Ethics Committee of Juntendo Urayasu Hospital on December 19th, 2017 (approval number: 29–075). This study was an observational study and did not involve any intervention, invasive examination, or treatment of the subject. As approved by the Ethics Committee of our hospital, we replaced the acquisition of individual consent by posting information about the research in the hospital and on the homepage to ensure the opportunity for patients to refuse the use of their data.

### 2.1. Study Subjects

We conducted a one-year observational study to investigate the risks of COPD exacerbation in the real-world data [15]. Patients who came to our department with a diagnosis of COPD in their medical records from January 1, 2018, to December 31, 2018, were recruited. Patients attending our department periodically as of January 1, 2018, were also recruited. The diagnosis of COPD was made by pneumonologists in our hospital based on the descriptions in the GOLD statement.

The exclusion criteria were as follows: spirometry not performed during the entry period and a one-year follow-up period. Patients with bronchiectasis and disease which cause eosinophilia, e.g., parasite infections and vasculitis, were also excluded. To examine the effect of inhalation therapy, patients who changed inhalation therapy or completed regular visits during the follow-up period were retrospectively traced from the date of change or end of the visit to ensure a one-year period of treatment.

### 2.2. Data Collection and Outcome

Patients' background information, including age, sex, smoking history, spirometry data, laboratory tests including blood eosinophil count, and comorbidities were collected while they were in a stable condition. Events of moderate to severe exacerbation defined by the GOLD statement during the one-year follow-up period were counted. Moderate exacerbation was defined as “treated with short-acting bronchodilators plus antibiotics and/or oral corticosteroids,” and severe exacerbation was defined as “patients required hospitalization or visits to the emergency room.” All patient data were collected from the electronic medical records of the hospital. Comorbidities of the respiratory system such as bronchial asthma and interstitial pneumonia were diagnosed by the doctors in charge of each patient according to Japanese guidelines.

### 2.3. Statistical Analysis

Continuous variables are shown as medians and ranges of minimum to maximum, and categorical variables are shown as numbers and percentages. To compare continuous variables, we used the Mann–Whitney *U* test, and Fisher's exact test was used to compare categorical variables. For the analysis of exacerbation risk factors, univariate and multivariate logistic regression analyses were performed, and the factors used for analysis were selected from the point of view of clinical importance. Two-sided *p* values <0.05 were considered statistically significant for all tests. All data analyses were performed using STATA 14 (STATA Corp., College Station, Texas, USA).

## 3. Results

### 3.1. Patient Background and Treatments

**Figure 1:**
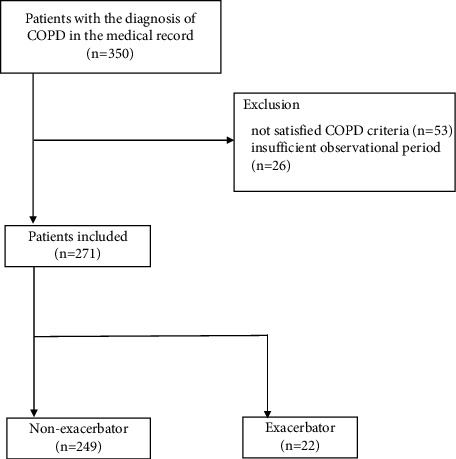
Flowchart of the patient selection. COPD, chronic obstructive pulmonary disease.

A total of 350 patients came to our hospital with a diagnosis of COPD in their medical records from January 1, 2018, to December 31, 2018. Among these, 53 patients were excluded because they did not meet the criteria of COPD at the point of spirometry: postbronchodilator FEV1/FVC% > 70% or no spirometry data; and 26 patients were further excluded because of the lack of follow-up phase for discontinuation of hospital visits and referral to other doctors. Eventually, 271 patients were enrolled and stratified based on whether they were nonexacerbators (*n* = 249, 91.9%) or exacerbators (*n* = 22, 8.1%) by the presence of moderate or severe exacerbation during the follow-up period (Figure 1).

Table 1 shows the comparison between nonexacerbators and exacerbators concerning patient background and comorbidities. Exacerbators were all male and consistently had worse pulmonary functions: %VC (*p*=0.036); FEV1 (*p*=0.015); FEV1/FVC (*p*=0.002); and % FEV1 (*p*=0.001). Blood eosinophil counts in the disease-stable period were higher in exacerbators, but the difference was not statistically significant (*p*=0.436). Comorbidities that may affect the choice of COPD treatment, such as benign prostatic hyperplasia (BPH), glaucoma, bronchial asthma (BA), and interstitial pneumonia (IP), were also compared, and there was no significant difference between the two groups. Therapies used during the study period are shown in Table 2.

For each component, ICS (*p*=0.028), long-acting *β*^2^-agonists (LABA, *p*=0.007), leukotriene receptor antagonists (LTRA, *p*=0.007), and home oxygen therapy (HOT) (*p* < 0.001) were used more frequently in exacerbators. In terms of combination use of inhalation therapy, no patient in the exacerbators group used long-acting muscarinic antagonist (LAMA) monotherapy, and triple inhalation therapy (ICS + LABA + LAMA) was used more frequently in exacerbators, with a borderline statistical difference (*p*=0.057).

### 3.2. Analysis of Factors Influential for Exacerbation

In current real-world data, we examined the factors that affected moderate or severe COPD exacerbation using univariate and multivariate analyses (Table 3).

Advanced age (>80; odds ratio [OR] = 2.74, 95% confidence interval [CI] 1.08–6.93, *p*=0.034), low %FEV1 (<50%; OR = 5.27, 95% CI 2.11–13.17, *p*=0.001), increased white blood cell count (>9000 ml; OR = 3.61, 95% CI 1.07–12.14, *p*=0.037), and HOT usage (OR = 6.49, 95% CI 2.51–16.76, *p* < 0.001) were associated with a high OR for exacerbations in the univariate analysis. High blood eosinophil count (≥350 cells (*μ*L); OR = 4.00, 95% CI 1.33–12.06, *p*=0.01), low %FEV1 (<50%; OR = 5.05, 95% CI 1.80–14.09, *p*=0.002), increased white blood cell count (>9000 ml; OR = 4.43, 95% CI 1.07–18.26, *p*=0.039) and HOT usage (OR = 4.86, 95% CI 1.63–14.50, *p*=0.005) were associated with a high OR for exacerbation events in the multivariate analysis.

### 3.3. Correlation of Blood Eosinophils and Exacerbation Risk

**Figure 2:**
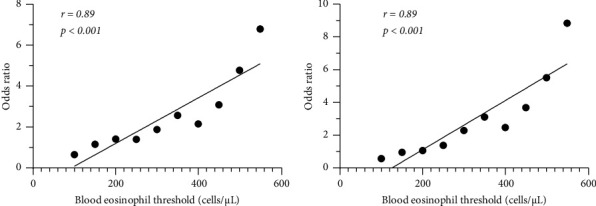
Correlation between blood eosinophil count and risk of exacerbation. (a) In all included patients; (b) excluding bronchial asthma patients. The solid line is the regression line.

We identified blood eosinophil count as one of the risk factors for COPD exacerbation (Table 3). Thus, we computed the OR using various cut-off values of blood eosinophils and analyzed the correlation between blood eosinophil counts and the OR for COPD exacerbation. There was a strong correlation between these parameters (Pearson correlation coefficient, *r* = 0.89, *p* < 0.001, Figure 2(a)). Even when excluding patients with medically recorded BA, we found the same trend (*r* = 0.89, *p* < 0.001, Figure 2(b)).

### 3.4. Inhalation Therapy and Exacerbation

We then examined the relationship between inhalation therapy and exacerbations; however, there was no significant correlation (Table 4).

Regarding the component of ICS, the risk of exacerbation increased (OR = 2.60, 95% CI, 1.07–6.31, *p*=0.035) in the univariate analysis but not in the multivariate analysis which was adjusted for COPD severity (OR = 1.68, 95% CI, 0.66–4.22, *p*=0.271). Since high blood eosinophils (≥350 cells/*μ*L) were revealed to increase the risk of exacerbation, we also analyzed inhalation therapy and the risk of exacerbation in patients with high (≥350 cells/*μ*L, *n* = 44, Table S1) and low eosinophil counts (<350 cells/*μ*L, *n* = 227, Table S2). Blood eosinophil levels did not affect the relationship between inhalation therapy and COPD exacerbation.

## 4. Discussion

The current study demonstrates the risk factors for COPD exacerbation in 271 COPD patients. We identified peripheral blood eosinophil count ≥350 cells/*μ*L, low %FEV1, increased white blood cell count, and HOT as risk factors for COPD exacerbation.

Several risk factors for COPD exacerbation have already been reported. Simultaneous elevation of C-reactive protein (CRP), fibrinogen, and white blood cell count has emerged as a risk factor for COPD exacerbation in a cohort study [21]. In another observational study, a history of exacerbation, history of gastroesophageal reflux or heartburn, worsening lung function, and poorer quality of life were found to be risk factors for COPD exacerbation. [22] Peripheral blood eosinophil count is suggested to be a predictor of COPD exacerbation [12, 13, 23]; however, some studies have shown no relationship between blood eosinophil count and COPD exacerbation [24]. As mentioned, 30%–40% of cases of COPD involve eosinophilic airway inflammation [8, 9], which was confirmed with eosinophils in sputum and bronchoalveolar lavage fluid [25, 26], and a correlation between blood and sputum eosinophils has also been demonstrated [11, 27, 28]. Eosinophilic airway inflammation is one of the COPD subtypes, and it causes COPD exacerbation from sources other than bacteria and viruses. Approximately 30% of COPD exacerbations can be classified as eosinophilic exacerbation [9]. Our data showed a strong positive correlation between peripheral blood eosinophil count and the risk of COPD exacerbation. Previous studies have determined cut-off values of blood eosinophils which were similar to our cut-off values (350 cells/*μ*L). [12–14] On the other hand, a post hoc analysis of pooled data from 11 clinical trials mentioned that peripheral blood eosinophil count is not significantly correlated with exacerbation risk, contrary to our results [18]. This discrepancy may be due to patient selection: clinical trials usually try to include pure-COPD patients and exclude patients with concomitant BA. The current study showed that the correlation between blood eosinophil count and the risk of exacerbation remained consistent, even in patients with BA. Therefore, we believe that peripheral blood eosinophil count is a useful predictor of COPD exacerbation, especially in a real-world setting.

In recent randomized control trials, triple inhalation therapy (ICS + LABA + LAMA) has been shown to reduce exacerbation [29, 30]. On the other hand, there is insufficient evidence for the benefits of ICS in COPD patients in real-world settings. Patients included in the current study had a relatively high tendency to use ICS, and ICS tended to be used more frequently in patients with severe COPD. As shown in Table 4, the current univariate analysis indicated that ICS usage may be associated with COPD exacerbation, but the multivariate analysis controlling for COPD severity did not. In the present study, the number of exacerbators was relatively small, which is a characteristic of COPD patients in Japan. This may have prevented adequate analyses of the data, especially in the multivariate analysis. We also performed the same analysis among high-eosinophil patients, which did not reveal a relationship between ICS usage and exacerbation, likely for the same reason (Table S1).

The current study has some limitations. Firstly, we recruited COPD patients from a single hospital which might have introduced selection bias and limited the number of patients. Secondly, the patient group in this study tended to have mild COPD compared to that in other studies [7, 20]. On the other hand, blood eosinophil levels in the patient cohort of this study were around 200 cells/*μ*L, similar to previous reports [31]. We have to consider that regional patient characteristics may have affected the results. In addition, we chose clinically important factors for the multivariate analysis; however, by including additional patients from multiple hospitals, it may be possible to collect more events of COPD exacerbations and analyze additional factors. Another limitation is the peripheral blood eosinophil stability. The stability of blood eosinophil counts remains a matter of debate. Blood eosinophils are sensitive to patient conditions and may easily shift above and below the cut-off value depending on the threshold. A previous study reported that 51% of patients were consistently above or below the cut-off value for blood eosinophils of ≥2% of whole white blood cells after a three-year follow-up period [31]. On the other hand, another study reported that 30–40% of admitted patients with acute COPD exacerbation moved above and below the cut-off value in three blood eosinophil records [7]. This problem may be resolved by setting a higher cut-off value which we did in the current study. Furthermore, we could not collect information about the history of previous exacerbations from the medical records. Because the history of previous exacerbation is a known exacerbation factor, there might be a bias in our study. High eosinophils were reported as a predictive factor of corticosteroid response and mortality in the patients with COPD exacerbations [32, 33]. In this study, we did not analyze the prognosis of the COPD exacerbation including the response to oral corticosteroid. Future studies should consider these factors. Finally, the choice of therapy depends on the physician in charge, so there might be additional confounders, which is a common feature of real-world data. Therefore, further research is needed, including more patients, to confirm real-world trends of COPD exacerbation.

## 5. Conclusions

Our data showed that peripheral blood eosinophil count is a good predictor of COPD exacerbation in a real-world setting. We can now prescribe triple inhalation therapy easily using single-inhaler devices; however, the peripheral blood eosinophil count in the clinical course of each COPD patient should be confirmed for proper management of this disease.

## Figures and Tables

**Table 1:** Comparison of patient characteristics and comorbidities between nonexacerbators and exacerbators.

	Nonexacerbators (*n* = 249)	Exacerbators (*n* = 22)	*p* value
*General information*			
Age	73 (52–92)	77 (51–89)	0.110
Male/female	219/30	22/0	0.068
Smoking history (Pack-year)	49 (0–220)	49 (20–96)	0.660
BMI (kg/m^2^)	22.1 (14.8–34.6)	21.7 (14.2–38.0)	0.560

*Examinations*			
VC (L)	3.04 (1.16–5.24)	2.81 (1.71–4.18)	0.139
%VC (%)	91.4 (40.4–154.1)	81.5 (53.2–125.1)	**0.036**
FEV_1_ (L)	1.61 (0.51–5.06)	1.31 (0.73–2.65)	**0.015**
FEV_1_/FVC (%)	58.48 (25.69–69.98)	46.39 (32.72–68.26)	**0.002**
%FEV_1_ (%)	64.6 (41.4–137.2)	45.6 (29.3–111.8)	**0.001**
Blood eosinophil (*μ*L)	180 (10–3210)	230 (40–1060)	0.436
WBC (*μ*L)	6250 (2300–15200)	6700 (3000–10400)	0.55
CRP (mg/dl)	0.2 (0.1–5.6)	0.25 (0.1–5.3)	0.20

*Comorbidities*			
HT	73 (29%)	7 (32%)	0.488
DM	30 (12%)	3 (14%)	0.518
BPH	45 (18%)	5 (22%)	0.382
Glaucoma	10 (4%)	1 (5%)	0.613
BA	33 (13%)	5 (23%)	0.178
IP	21 (8%)	1 (5%)	0.448

*Note.* Data are shown as median (range). Significant differences are marked using the bold font. BMI, body mass index; VC, vital capacity; %VC, VC/predicted VC; FEV1, forced expiratory volume in 1 s; FVC, forced vital capacity; %FEV1, FEV1/predicted FEV1; WBC, white blood cell; CRP, C-reactive protein; HT, hypertension; DM, diabetes mellitus; BPH, benign prostatic hyperplasia; BA, bronchial asthma; IP, interstitial pneumonia.

**Table 2:** Comparison of therapies between nonexacerbators and exacerbators.

	Nonexacerbators (*n* = 249)	Exacerbators (*n* = 22)	*p* value
*Component included*			
ICS	89 (36%)	13 (59%)	**0.028**
LABA	197 (79%)	22 (100%)	**0.007**
LAMA	179 (72%)	14 (64%)	0.277

*Inhalation therapy*			
ICS mono	1 (0.4%)	0 (0%)	0.919
LABA mono	22 (9%)	4 (18%)	0.146
LAMA mono	31 (12%)	0 (0%)	0.061
ICS + LABA	29 (12%)	4 (18%)	0.272
LAMA + LABA	89 (36%)	5 (23%)	0.160
ICS + LABA + LAMA	57 (23%)	9 (41%)	0.057

*Others*			
Macrolide	32 (13%)	5 (23%)	0.164
Theophylline	23 (9%)	5 (23%)	0.061
LTRA	18 (7%)	6 (27%)	**0.007**
HOT	24 (10%)	9 (41%)	**<0.001**

*Note.* Data are shown as number of patients (percentage). Significant differences are marked using bold font. Macrolide and LTRA were administered orally. ICS, inhaled corticosteroid; LABA, long-acting *β*^2^-agonist; LAMA, long-acting muscarinic antagonist; LTRA, leukotriene receptor antagonist; HOT, home oxygen therapy.

**Table 3:** Univariate and multivariate analysis of the exacerbation risk factor.

	Univariate				Multivariate	
	Odds ratio (95% CI)		*p* value		Odds ratio (95% CI)	*p* value
Age	<60	2.54 (0.78–8.24)		0.120	—	—
	60∼70	0.13 (0.01–1.04)		0.055	—	—
	70∼80	0.72 (0.29–1.74)		0.468	—	—
	≥80	2.74 (1.08–6.93)		**0.034**	—	—

BMI	<18.5	1.27 (0.35–4.59)		0.710	—	—
	18.5∼25	1.10 (0.41–2.95)		0.835	—	—
	≥25	0.70 (0.19–2.47)		0.582	—	—
Smoking history	Pack-year ≥ 50	1.03 (0.43–2.47)		0.952	—	—

%FEV1	≥80%	0.48 (0.13–1.70)		0.259	—	—
	50%∼80%	0.28 (0.10–0.78)		**0.016**	—	—
	<50%	5.27 (2.11–13.17)		**<0.001**	5.05 (1.80–14.09)	**0.002**

%VC	<70%	1.99 (0.68–5.77)		0.203	—	—
	70%∼80%	0.90 (0.25–3.21)		0.877	—	—
	80%∼100%	1.26 (0.52–3.03)		0.602	—	—
	≥100%	0.46 (0.15–1.40)		0.173	—	—

Blood eosinophil	<150 (*μ*L)	0.86 (0.34–2.13)		0.750	—	—
	150∼350 (*μ*L)	0.57 (0.22–1.46)		0.244	—	—
	≥350 (*μ*L)	2.57 (0.98–6.74)		0.054	4.00 (1.33–12.06)	**0.014**
WBC	≥9000 (*μ*L)	3.61 (1.07–12.14)		**0.037**	4.43 (1.07–18.26)	**0.039**
CRP	≥0.3 mg/dl	1.43 (0.57–3.59)		0.439	—	—

Comorbidity	HT	1.12 (0.44–2.87)		0.805	—	—
	DM	1.15 (0.32–4.12)		0.827	—	—
	BA	1.93 (0.67–5.57)		0.227	—	—
	IP	0.52 (0.07–4.04)		0.529	—	—
	HOT	6.49 (2.51–16.76)		**<0.001**	4.86 (1.63–14.50)	**0.005**

%FEV1 <50%, blood eosinophil ≥350 (*μ*L), WBC >9000 (*μ*L), and HOT were included in the multivariate analysis. Statistical significance is marked using the bold font. FEV1, forced expiratory volume in 1 s; WBC, white blood cell; CRP, C-reactive protein; HT, hypertension; DM, diabetes mellitus; BA, bronchial asthma; IP, interstitial pneumonia; HOT, home oxygen therapy.

**Table 4:** Univariate and multivariate analyses of inhalation therapy and exacerbation in all patients.

	Univariate		Multivariate	
	Odds ratio (95% CI)	*p* valu*e*	Odds ratio (95% CI)	*p* value
*Component included*				
ICS	2.60 (1.07–6.31)	**0.035**	1.68 (0.66–4.22)	0.271
LAMA	0.68 (0.28–1.70)	0.415	0.56 (0.21–1.49)	0.253
LABA	N.D	N.D	N.D	N.D

*Inhalation therapy*				
ICS mono	N.D	N.D	—	—
LAMA mono	N.D	N.D	—	—
LABA mono	2.29 (0.71–7.38)	0.164	—	—
ICS + LABA	1.69 (0.53–5.33)	0.374	2.41 (0.54–10.73)	0.247
LAMA + LABA	0.53 (0.19–1.48)	0.225	0.87 (0.21–3.47)	0.847
ICS + LABA + LAMA	2.33 (0.95–5.73)	0.065	2.04 (0.57–7.30)	0.269

ICS, LAMA, LABA, and ICS + LABA, LAMA + LABA, and ICS + LABA + LAMA were included in each multivariate analysis. The analysis was adjusted for COPD severity. Statistical significance is marked using the bold font. ICS, inhaled corticosteroid; LAMA, long-acting muscarinic antagonist; and LABA, long-acting *β*^2^-agonist.

## Data Availability

Data are made available upon reasonable request to the corresponding author.

## Supplementary Materials

Supplementary Materials Table S1: Univariate and multivariate analyses of inhalation therapy and exacerbation in patients with eosinophils ≥350 cells/*μ*L. Table S2: Univariate and multivariate analysis of inhalation therapy and exacerbation in patients with eosinophils <350 cells/*μ*L.
