# Supplementary material for: Blood Eosinophil Count as a Predictive Biomarker of Chronic Obstructive Pulmonary Disease Exacerbation in a Real-World Setting
Source: Can Respir J. 2023 May 25;2023:3302405. doi: 10.1155/2023/3302405 (PMC10234729; doi:10.1155/2023/3302405)
Supplement: Supplementary Materials — Table S1: Univariate and multivariate analyses of inhalation therapy and exacerbation in patients with eosinophils ≥350 cells/μL. Table S2: Univariate and multivariate analysis of inhalation therapy and exacerbation in patients with eosinophils <350 cells/μL. [file 3302405.f1.docx]

**Table S1.** Univariate and multivariate analysis of inhalation therapy and exacerbation in patients with eosinophils ≥ 350 cells/μL

|  | Univariate | | Multivariate | |
| --- | --- | --- | --- | --- |
|  | Odds ratio (95% CI) | *p-value* | Odds ratio (95% CI) | *p-value* |
| Component included | | | | |
| ICS | 3.67 (0.63-21.45) | *0.149* | 2.77 (0.46-16.74) | *0.268* |
| LAMA | 0.93 (0.15-5.56) | *0.933* | 0.71 (0.10-4.77) | *0.721* |
| LABA | N.D. | N.D. | N.D. | N.D. |
| Inhalation therapy | | | | |
| ICS mono | N.D. | N.D. | - | - |
| LAMA mono | N.D. | N.D. | - | - |
| LABA mono | 2.92 (0.23-37.43) | *0.411* | - | - |
| ICS+LABA | 1.38 (0.13-14.53) | *0.791* | 2.25 (0.11-45.72) | *0.598* |
| LAMA+LABA | 0.27 (0.30-2.52) | *0.253* | 0.64 (0.036-11.63) | *0.765* |
| ICS+LABA+LAMA | 3.60 (0.68-19.00) | *0.131* | 3.60 (0.34-38.48) | *0.289* |

Abbreviations: ICS, inhaled corticosteroid; LAMA, long-acting muscarinic antagonist; LABA, long-acting β2-agonist

ICS, LAMA, LABA, and ICS+LABA, LAMA+LABA, ICS+LABA+LAMA were included in each multivariate analysis.

**Table S2.** Univariate and multivariate analysis of inhalation therapy and exacerbation in patients with eosinophils < 350 cells/μL

|  | Univariate | | Multivariate | |
| --- | --- | --- | --- | --- |
|  | Odds ratio (95% CI) | *p-value* | Odds ratio (95% CI) | *p-value* |
| Component included | | | | |
| ICS | 2.13 (0.74-6.11) | *0.159* | 1.40 (0.48-4.11) | *0.535* |
| LAMA | 0.59 (0.20-1.74) | *0.339* | 0.58 (0.19-1.75) | *0.335* |
| LABA | N.D. | N.D. | N.D. | N.D. |
| Inhalation therapy | | | | |
| ICS mono | N.D. | N.D. | - | - |
| LAMA mono | N.D. | N.D. | - | - |
| LABA mono | 2.40 (0.62-9.22) | *0.202* | - | - |
| ICS+LABA | 1.87 (0.49-7.09) | *0.357* | 2.60 (0.49-13.75) | *0.261* |
| LAMA+LABA | 0.66 (0.20-2.16) | *0.496* | 1.16 (0.25-5.35) | *0.853* |
| ICS+LABA+LAMA | 1.76 (0.57-5.39) | *0.325* | 2.30 (0.52-10.12) | *0.269* |

Abbreviations: ICS, inhaled corticosteroid; LAMA, long-acting muscarinic antagonist; LABA, long-acting β2-agonis

ICS, LAMA, LABA, and ICS+LABA, LAMA+LABA, ICS+LABA+LAMA were included in each multivariate analysis.
